# Resolution of insulin resistance, lactic acidosis, and decrease in mechanical support requirements in patients post orthotopic heart transplant with the use of long-acting insulin glargine

**DOI:** 10.1186/s13019-024-02543-y

**Published:** 2024-02-16

**Authors:** Ribal Darwish, Eva Chen, Steven Minear, Cedric Sheffield

**Affiliations:** 1 Anesthesiology Institute, Surgical Critical Care Division, Cleveland Clinic Florida, Weston, USA; 2 Department of Thoracic and Cardiovascular Surgery, Cleveland Clinic Florida, Weston, USA

**Keywords:** Insulin resistance, Lactic acidosis, Orthotopic heart transplant

## Abstract

**Objective:**

This study investigates the efficacy of using a long-acting insulin analog, along with the infusion of regular insulin, in achieving appropriate glycemic control and correcting lactic acidosis in patients post orthotopic heart transplant who demonstrate severe lactic acidosis and insulin resistance.

**Methods:**

This was a retrospective study of two cohorts (IRB FLA 20-003) of patients post orthotopic heart transplant with severe lactic acidosis and insulin resistance who were admitted to a tertiary intensive care unit and treated with (group 1) or without long-acting insulin analog (group 2) within the first 24 h of admission to the intensive care unit. Insulin resistance is defined as the requirement for intravenous regular insulin infusion of more than 20 units/h without the ability to achieve appropriate serum glucose level (120–180 mg /dL). Severe lactic acidosis is defined as arterial lactic acid of more than 10 mmol/L. The following parameters were investigated: time to correct lactic acidosis, duration of postoperative mechanical ventilation, the need for periprocedural mechanical circulatory support, and 28-day mortality.

**Results:**

The 28-day mortality was zero in both groups. Two patients required periprocedural mechanical support in group one, and ten patients required mechanical support in group two (RR = 0.224, 95%, confidence interval 0.052–0.95, Z = 2.029, p = 0.042). Three patients required tracheostomy in group one, and four patients required tracheostomy in group two (RR 0.84, 95 confidence interval 0.20–3.48, Z = 0.23, P = 0.81). Wilcoxon rank-sum test was used to compare time to correct lactic acidosis, with lactic acid resolution being faster in group one ($\overline{X}$_1_ = 19.7 h, SD ± 12.6 h $\overline{X}$_2_ = 29.3 h, SD ± 19.6 h, Z-value − 2.02, p-value 0.043). The duration of mechanical ventilation was less in group one ($\overline{X}$_1_ = 29 h, SD ± 12.7 h, $\overline{X}$_2_ = 55.1 h, SD ± 44.5 h, Z-value: − 1.92, p-value 0.05).

**Conclusion:**

Administration of low-dose long-acting insulin glargine led to the resolution of the lactic acidosis, insulin resistance, and decreased requirements for pressor and inotropic support, which led to decreased need for mechanical circulatory support.

## Introduction

Glycemic control is an essential component of periprocedural care for patients status post heart transplantation. Both acute and chronic insulin resistance lead to transplant graft malfunction, increased need for pressor and inotropic support and fluid resuscitation. The standard practice in intensive care is to use intravenous infusion of regular insulin. This practice has been promoted for its ease of titration and the short half-life of the regular insulin. Unfortunately, in many situations post heart transplantation, patients develop insulin resistance, lactic acidosis, and high requirements for epinephrine infusion. We reviewed the efficacy of using long-acting insulin analog, along with the infusion of regular insulin, in achieving glycemic control in patients with severe lactic acidosis and insulin resistance. Additionally, we examined if rapid correction of lactic acidosis leads to decreased epinephrine infusion requirement, time to extubation, and 28-day mortality, as well as the need for postoperative mechanical support.

## Study design, setting, and participants

This was a retrospective study of two cohorts of patients post orthotopic heart transplant with severe lactic acidosis and insulin resistance who were admitted to a tertiary intensive care unit treated with (33 patients) or without long-acting insulin analog (37 patients) within the first 24 h of intensive care unit admission. There were 57 males and 13 females in this study. The average age was 57.5 years. In group one, 42% of the patients had diagnosis of type II diabetes mellitus, compared to 35% in group two. Insulin resistance was defined as insulin infusion administration at a dose of at least 20 units/h to achieve serum glucose level of 120–180 mg/dL, and severe lactic acidosis was defined as lactic acid of more than 10 mmol/L. All patients were managed based on a standard postoperative protocol with the goal to maintain cardiac index $\ge$ 3 L/min/ M^2^, CVP: (8–10) cm.H_2_O, PADP: (12–16) cm.H_2_O, SVO2 $\ge$ 60%. On arterial blood gas analysis, lactic acid was measured every 4 h. Glycemic control was achieved by using an infusion of regular insulin with goal serum glucose 120–180 mg/dl per institutional protocol. The following parameters were analyzed: time to correct lactic acidosis, duration of postoperative mechanical ventilation, the need for periprocedural mechanical support, and 28-day mortality.

## Results

Seventy patients were reviewed, all of whom met the criteria for perioperative insulin resistance Table 1. 33 patients were treated with glargine in addition to regular insulin infusion (group one), and 37 were treated with intravenous insulin infusion only (group two). The 28-day mortality was zero in both groups. Two patients required periprocedural mechanical circulatory support in group one, and ten patients required mechanical support in group two (RR = 0.224, 95%, confidence interval 0.052–0.95, Z = 2.029, p = 0.042). Three patients required tracheostomy in group one, and four patients required tracheostomy in group two (RR 0.84, 95 confidence interval 0.20–3.48, Z = 0.23, P = 0.81). Wilcoxon rank-sum test was used to compare time to correct lactic acidosis; lactic acid resolution was faster in group one ($\overline{X}$_1_ = 19.7 h, SD ± 12.6 h, $\overline{X}$_2_ = 29.3 h, SD ± 19.6 h, Z-value − 2.02, p-value 0.043). The duration of mechanical ventilation was less in group one ($\overline{X}$_1_ = 29 h, SD ± 12.7 h, $\overline{X}$_2_ = 55.1 h, SD ± 44.5 h, Z-value: − 1.92, p-value 0.05).

**Table 1:** Outcome data

	Group 1	Group 2	
Time to correct lactic acidosis (h)	$\bar{X}_{1}$ = 19.7 ± 12.6	$\bar{X}_{2}$ = 29.3 ± 19.6	Z-value = − 2.02, p-value = 0.04
Time to extubation (h)	$\bar{X}_{1}$ = 29 ± 12.7	$\bar{X}_{2}$ = 55.1 ± 44.5	Z-value = − 1.92, p-value = 0.05
Periprocedural requirement for IABP (N)	2/33	10/37	RR = 0.22, 95% Cl = 0.05–0.95, Z = 2.02, p = 0.04
Requirement for tracheostomy (N)	3/33	4/37	RR = 0.84, 95% Cl = 0.20–3.48, Z = 0.23, p = 0.81

Group 1 consists of patients treated with insulin analog in addition intravenous insulin infusion. Group 2 consists of patients treated with intravenous insulin infusion only

**Fig. 1:**
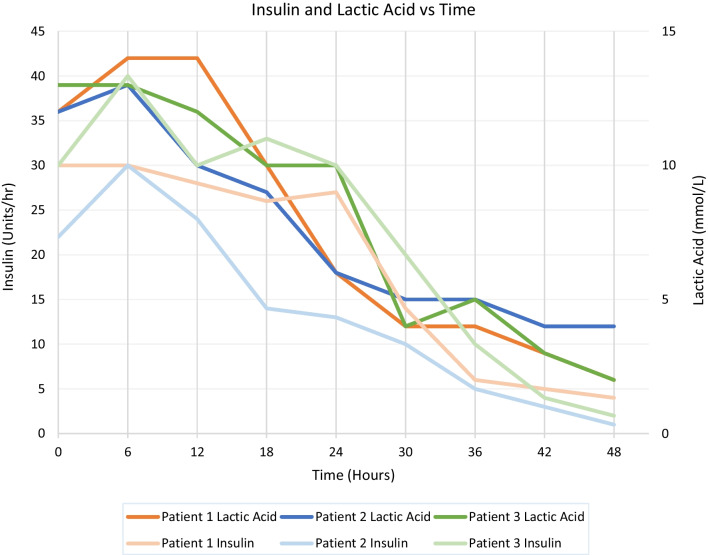
The timeline resolution of insulin resistance after administration of insulin analog in three patients with refractory insulin resistance

The timeline resolution of insulin resistance after administration of insulin analog in three patients with refractory insulin resistance Fig. 1.

## Discussion

Appropriate glycemic control in patients post open heart surgery leads to a lower incidence of sternal wound infection [1] and decreased mortality [2]. Insulin therapy is an essential component of periprocedural care post heart transplantation, as acute and chronic insulin resistance both lead to graft malfunction. Heart failure is known to coexist with type II diabetes mellitus, and this remains true for all heart failure patients including those with low or preserved left ventricular systolic function [3]. Despite the relationship between insulin resistance and heart failure, there currently exists no diagnostic criteria or guidelines for treatment. Moreover, the concept of myocardial insulin resistance is not well studied and difficult to diagnose in clinical practice.

Multiple studies have shown diabetes mellitus often coexists with heart failure, suggesting that these pathologies exacerbate each other clinically. In heart failure patients who receive mechanical circulatory support, insulin resistance improved [4]. It has been shown that activation of B2AR (beta-2 adrenergic receptors) regulates cardiac glucose uptake and promotes insulin resistance, a condition that was reported in heart failure. Insulin stimulation of B2AR promotes coupling with Gi and expression of phosphodiesterase 4 which compromise cardiac contractility [5–7]. This cycle in which activation of B2AR leads to insulin resistance and impaired cardiac function increases the requirement for inotropic support [8].

Insulin glargine (Lantus) is a long-acting human insulin analog that is used in patients in type I and type II diabetes. Insulin glargine differs from human insulin in that the amino acid asparagine at A21 is replaced by glycine and two arginines are added to the C-terminus of the B-chain. In our cohort, infusion of large doses of regular insulin did not lead to adequate glycemic control or correction of lactic acidosis. The addition of low dose long acting-insulin analog led to the resolution of the lactic acidosis, significant decrease in the dose of infused regular insulin, as well as decrease of the required epinephrine dose.

The timeline of this resolution matches the timing of long-acting insulin analog administration. The effect starts 4 h post subcutaneous infusion and lasts for 24 h. Within 4 h of administration of insulin analog, insulin requirements begin to decline dramatically to achieve our goal serum glucose of 120–180 mg/dL. Within a few hours, epinephrine infusion was titrated to maintain the standard cardiac index goal, which was associated with normalization of the lactic acid. Numerous etiologies are responsible for the periprocedural lactic acidosis post heart transplant, including circulatory failure, regional tissue ischemia, sepsis with liver being the primary organ responsible for lactate clearance. The resolution of lactic acidosis is dependent on the resolution of the underlying pathology.

In our series the rapid resolution of lactic acidosis was predictable post administration of insulin analog. As per our standard postoperative protocol, patient’s hemodynamics were managed to maintain cardiac index $\ge$ 3L/min/M^2^, the inotropic support and volume management are titrated to achieve this goal. Interestingly after the correction of the lactic acidosis the epinephrine infusion requirements decreased significantly, this pattern of recovery indicated that the initial event was the correction of the lactic acidosis by most likely the modulation of beta-adrenergic receptors and that subsequently lead to decrease of epinephrine dose infusion requirements. Figure 1 presents the timeline resolution of lactic acidosis of sample of three patients with insulin resistance and type II diabetes mellitus after administration of long-acting insulin analog post orthotopic heart transplant.

Currently, due to the benefit of adding long-acting insulin analog, it is an accepted practice in our institution to add glargine along with the intravenous insulin infusion to patients with insulin resistance and severe lactic acidosis. In patients without episodes of hypoglycemia, we caution that the required intravenous insulin dose will decrease after the administration of the insulin analog. Based on efficacy compared to regular insulin (one to one), the administration of low dose glargine is not comparable to the very large doses of the infused regular insulin. One possible explanation is that the long-acting insulin analog normalizes the B2AR receptor activation, where regular insulin performs in normal fashion.

Similar observations have been reported in non-critically ill patients with type II diabetes mellitus, in whom the level of HOMA-insulin resistance index (HOMA-IR) in the insulin-glargine group was significantly lower than that observed in the standard-care group [9]. These findings in heart transplant patients are more profound because cardiac denervation, along with the loss of the presynaptic neuronal uptake, leads to hypersensitivity to uptake-1 dependent catecholamines [10]. The preoperative diagnosis of diabetes mellitus type II has been identified as a risk factor for periprocedural insulin resistance and severe lactic acidosis. The difference between the cohort groups could be attributed to the fact that prolonged state of severe lactic acidosis may be subject to more aggressive fluid resuscitation, which could contribute to prolonged post-operative requirements for ventilatory and inotropic support. These findings must be further explored in a randomized trial, as the implications of these findings could affect the management of insulin resistance in patients with HF.

## Conclusion

Adding low-dose long-acting insulin glargine to the insulin infusion had facilitated the resolution of the lactic acidosis, insulin resistance and decreased requirements for pressor and inotropic support in heart transplant patients with insulin resistance, which led to the decrease in the periprocedural requirements for mechanical support.

## Data Availability

The data is saved on the Cleveland Clinic Computer system.
